# m6A-Related lncRNA Signature Is Involved in Immunosuppression and Predicts the Patient Prognosis of the Age-Associated Ovarian Cancer

**DOI:** 10.1155/2022/3258400

**Published:** 2022-08-10

**Authors:** Ming Li, Liang Zhang, Miaoxiao Feng, Xiao Huang

**Affiliations:** Department I of Obstetrics and Gynecology, Cangzhou Central Hospital, Cangzhou, China

## Abstract

**Background:**

Epithelial ovarian cancers are age-associated diseases, usually diagnosed at an advanced stage. lncRNA has been discovered to interplay with N6-methyladenosine (m6A), working in tandem to promote cancer progression and worsening patient outcomes. This study is aimed at investigating the roles and mechanism of m6A-related lncRNA signature on ovarian cancers.

**Methods:**

We retrieved TCGA and CGGA sequencing data to identify m6A-related lncRNA signature and constructed an m6A score (MS) using the LASSO algorithm. A clinical nomogram was then established to predict the overall survival of patients. Subsequently, GSEA analyses were conducted to obtain pathways involved. Expression of HLA genes, 28 tumor-infiltrating lymphocyte infiltration, and anticancer cycle were analyzed the immunological differences between high-MS and low-MS groups. Finally, immune checkpoint gene expressions and IC_50_ of chemotherapeutic drugs were calculated, and CMap was run to identify the potential compounds and their corresponding mechanisms.

**Results:**

We identified 16 m6A-related lncRNAs and constructed an MS model. The high-MS group showed a poor prognosis. A clinical nomogram consists of MS, and age was constructed and predicted the 1-, 3-, and 5-year survival with high accuracy. GSEA analyses presented downregulated antigen processing and presentation pathways. Immunocyte infiltrating analyses demonstrated that high-MS was associated with high infiltration of Treg cells, macrophages, and low Th1/Th2 rate. Also, high expression of immune checkpoint genes *NRP1*, *TNFSF9*, and *VSIR* was observed in the high-MS group. Finally, the high-MS group also predicted low IC_50_ of vinorelbine and vorinostat.

**Conclusion:**

This study constructed a robust prediction model for prognostic management and revealed the cross-talk between m6A and immunosuppression. Besides, the m6A lncRNA signature can predict the chemotherapeutic drug response. These will shed light on the development of novel therapeutic strategies and render survival benefits for ovarian patients.

## 1. Introduction

Ovarian cancer is one of the most predominant gynecological diseases in the world, containing a highly heterogeneous group of malignant tumors in etiology and genetic features. The largest proportion of ovarian cancer is epithelial, presenting an advanced stage at the time of first diagnosis [1] and higher mortality rates in aged patients [2, 3]. Serous carcinomas are the most common epithelial ovarian cancer; their routine treatments for serous ovarian cancers are surgical resection and adjuvant therapy. 51% of serous carcinoma patients are diagnosed at stage III, and 29% are diagnosed at stage IV, with low 5-year cause-specific survival of 42% and 26%, respectively [4]. Apart from the ineffective early screening, the poor prognosis also lies in the frequent recurrence and resistance to chemotherapy [5]. However, the disease-specific genetic aberrance has allowed for the effectiveness of targeted treatment. Also, individual genetic detection contributed to the prognostic management of serous ovarian cancer patients [6]. Cancer prediction models were construed for individualized diagnosis or prognosis estimation; previous models comprised clinical or anatomical predictors, which presented limited predictive accuracy, while models incorporating gene panels presented higher performances [7]. On top of that, high-quality genetic models are urgently required.

N6-methyladenosine (m6A) has emerged as the most common modification of RNA; its reversible functions were mediated by three types of functional components: “writers,” “readers,” and “erasers.” The “writers” were complexes of methyltransferases, such as METTL3 and METTL14, adding m6A on RNAs, while the “erasers,” like the demethylase FTO, removes the m6A away. Some specific RNA binding proteins (“readers”) can recognize the m6A and affect the fate of RNAs, including YTHDC1 and IGF2BPs [8]. Long noncoding RNA (lncRNA) has been found to interact with m6A in various malignant cancers [9]. For instance, the m6A “reader” can be stabilized by lncRNA and promoted the proliferation of cancers by downstream regulation [10]. m6A modification can also be exerted on lncRNA and affect the phenotypes of cancers [11, 12]. This interplay between lncRNA and m6A demonstrated the critical role of lncRNA in the cancer m6A-induced processes.

m6A-related lncRNA was engaged in multiple biological processes affecting cancer progress [13]. Recently, it was found to interplay with immune activities in many cancers; these participations included its roles in affecting immune response, tumor microenvironment remodeling, and response to immune checkpoint inhibitor therapy [14–16]. However, the cross-talk between m6A and immune remains elusive.

Here, we identified m6A-related lncRNA to construct a LASSO-based prognostic model called m6A score (MS), for predicting the survival of ovarian cancer patients. The roles of MS signature in cancer immune and drug sensitivities were also explored in multiple levels. This study offers a robust prediction tool in patient prognostic management and sheds light on the cross-talk between m6A and immune-related cancer properties. This will trigger novel therapeutic strategies targeting the critical m6A-related lncRNAs and improve the prognosis of ovarian cancer patients.

## 2. Material and Method

### 2.1. Patient Datasets and lncRNA Sets

The clinical features, RNA expression, copy number variation (CNV), and single nucleotide polymorphism (SNP) data of The Cancer Genome Atlas (TCGA) samples with *n* = 377 were retrieved via the R package “TCGAbiolinks” from https://www.cancer.gov/about-nci/organization/ccg/research/structural-genomics/tcga. The Gene Expression Omnibus (GEO) sample datasets GSE26193 (*n* = 107) were obtained using the R package “GEOquery” from https://www.ncbi.nlm.nih.gov/geo/. All expression data were transferred to transcripts per million (TPM) for further analyses. TCGA dataset was assigned as the training set, and the GEO dataset was used for independent validation. A total of 4183 lncRNAs were extracted from TCGA RNA matrix.

### 2.2. Construction of the m6A Score via the m6A-Related lncRNA Signature

We first selected the 23 m6A-related regulators as symbols of m6A modification [17], including *CBLL1*, *VIRMA*, *METTL14*, *METTL3*, *RBM15*, *RBM15B*, *WTAP*, and *ZC3H13* as writers; *ALKBH5* and *FTO* as erasers; and *ELAVL1*, *FMR1*, *HNRNPA2B1*, *HNRNPC*, *IGF2BP1*, *IGF2BP2*, *IGF2BP3*, *LRPPRC*, *YTHDC1*, *YTHDC2*, *YTHDF1*, *YTHDF2*, and *YTHDF3* as readers. The lncRNAs were filtered according to their correlations with the 23 m6A regulators; those lncRNAs with absolute correlation coefficients less than 0.3 were removed. Subsequently, the lncRNAs passing the filtration were input to the univariate Cox hazard analysis for identifying the survival-associated lncRNAs, and the least absolute shrinkage and selection operator (LASSO) algorithm [18] was performed to select the candidate predictors to construct the m6A score (MS). The MS was then constructed according to the formula below:

(1) $$\begin{array}{c} \text{m}6\text{A Score}\left(\text{MS}\right)=\overset{n}{\underset{i}{\sum }}\beta_{i}*l_{i}, \end{array}$$

where *l*_*i*_ refers to the expression level of the lncRNA *i* and *β*_*i*_ means the calculated coefficients of lncRNA *i* from the LASSO algorithm. Their corresponding coefficients were visualized in a lollipop chart.

### 2.3. Survival Prediction and the Performance Estimation of the MS

The training set and validating set were divided into high-MS and low-MS groups according to the median value of the m6A score. Kaplan-Meier curves were plotted to verify whether the MS can separate the overall survival (OS), progression-free interval (PFI), and disease-specific survival (DSS) rate of the two groups. Further, receiver operating characteristic curve (ROC) was performed to estimate the accuracy of the MS for 1-, 3-, and 5-year OS prediction.

### 2.4. Correlation between Copy Number Variation, Single Nucleotide Polymorphism, and MS

The downloaded SNP and CNV data were organized to compare the differences between high-MS and low-MS groups. CNV includes amplifications and deletions among the genomic region; the two CNV events were measured by GSITIC 2.0 [19]. Besides, we used the R package “matfool” [20] to analyze the driving mutation gene in the two groups.

### 2.5. Establishment of a Clinical Nomogram

The MS as well as the clinical factors (including diagnosis age, tumor stage, and grade) was analyzed by univariate Cox regression and multivariate Cox regression successively to obtain the independent parameters for establishing a quantitative prediction nomogram [21]. The nomogram was then assessed by calibration curves to measure the consistency between the predicted and actual probability of 1-, 3-, and 5-year OS [22].

### 2.6. Functional Analyses for the lncRNA Signature

To discover the biological mechanisms of the lncRNA signature functions, we seek the pathways on which the lncRNA signature was enriched. Gene set variation analysis (GSVA) [23] was performed on the high-MS and low-MS groups. The GSVA depicted the hallmark pathway enrichment differences between the two groups visualized in a heatmap; the hallmark gene sets (h.all.v7.4.entrez.gmt) were downloaded from Molecular Signatures Database (MSigDB, http://software.broadinstitute.org/gsea/msigdb). We then conducted GSEA [24] to calculate the enrichment score of the positively and negatively enriched KEGG pathways (c2.cp.kegg.v7.4.entrez.gmt) [25] from MSigDB.

### 2.7. The Stromal and Immune Infiltration in the Tumor Environment

The tumor microenvironment contains various stromal cells and immunocytes. We estimated the stromal infiltrating via calculation of stromal score and tumor purity by Estimation of STromal and Immune cells in MAlignant Tumours using Expression data (ESTIMATE) [26]. Besides, the leukocyte infiltration level differences between high-MS and low-MS groups were compared using the 24 HLA markers (*HLA-E*, *HLA-DPB2*, *HLA-C*, *HLA-J*, *HLA-DQB1*, *HLA-DQB2*, *HLA-DQA2*, *HLA-DQA1*, *HLA-A*, *HLA-DMA*, *HLA-DOB*, *HLA-DRB1*, *HLA-H*, *HLA-B*, *HLA-DRB5*, *HLA-DOA*, *HLA-DPB1*, *HLA-DRA*, *HLA-DRB6*, *HLA-L*, *HLA-F*, *HLA-G*, *HLA-DMB*, *HLA-DPA1*).

### 2.8. Immunocyte Infiltration and Anticancer Cycle Analysis

To clarify the immune landscape, we explore the immunocyte infiltration via analysis of 28 tumor-infiltrating lymphocytes (TILs) [27] on The Cancer Immunome Atlas (https://tcia.at/). The coefficients of the correlations between the MS and 28 TILs were calculated, and their correlations were visualized in the correlation plot. Subsequently, the correlation between MS and the antitumor cell cycle [28] was calculated; this cycle was defined as 7 steps, including the release of cancer cell antigens (step 1), antigen presentation (step 2), priming and activation of immunocytes (step 3), trafficking of immunocytes to tumors (step 4), infiltrating of immunocytes into tumors (step 5), recognition of cancer cell (step 6), and killing of cancer cells (step 7). The coefficient calculation of the correlations between MS and each step of the cycle manifested the roles of MS in antitumor activity.

### 2.9. Therapeutic Estimation of Immune Checkpoint and Chemotherapy Drugs

The immune checkpoint mediates the suppression of lymphocyte activation and escapes cancers from immune supervision. To investigate whether immune checkpoints play roles in the procancer activities, we compared the expression level of PD-1, CTLA4, and 14 immune checkpoint genes between high-MS and low-MS groups. We also estimated the predicted half-maximal inhibitory concentration (IC_50_) of commonly applied chemotherapy drugs for obtaining the drug sensitivities of ovarian cancer patients, including cisplatin, doxorubicin, gemcitabine, paclitaxel, vinorelbine, and vorinostat. Finally, we screened candidate chemical compounds targeting the m6A features based on the 202 differentially expressed genes identified between high-MS and low-MS patients; this analysis was performed by the mechanism of actions (MoA) among those compounds using CMap tools (https://clue.io/) [29].

### 2.10. Statistical Analyses

The statistical analyses were conducted in the R software (version 3.6.3). A two-sided *p* value < 0.05 was regarded as statistically significant. Log-rank test was used for the Kaplan-Meier curves of TCGA and GEO dataset patient survival analyses. For normally distributed variables, we used Student's *t*-test to conduct the pairwise comparisons, and for nonnormally distributed variables, the Wilcoxon test was performed. Euclidean distance adopted in hierarchical cluster analysis was applied to the heatmap graphing. Finally, Spearman correlation analysis was used to compute the significance of correlations between variables; absolute correlation coefficients > 0.3 were considered as correlated. For the symbols, ∗∗∗, ∗∗, ∗, and NS refer to *p* < 0.001, <0.01, <0.05, and not significant, respectively [30].

## 3. Results

### 3.1. Identification of a 16 N6-Methyladenosine-Associated lncRNA m6A Score via LASSO Regression

**Figure 1:**
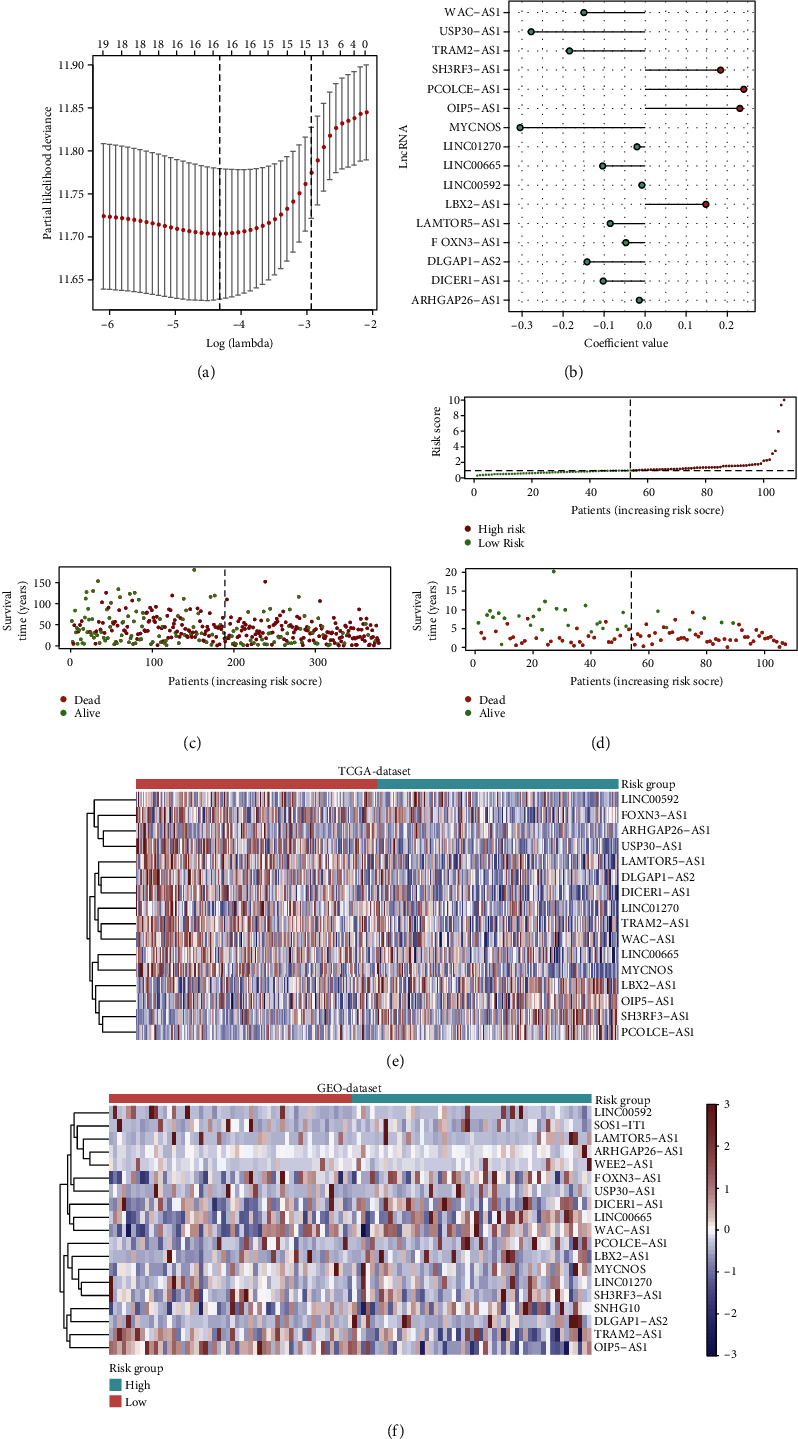
Identification of a 16 N6-methyladenosine-associated lncRNA m6A score via LASSO regression. (a) LASSO regression. (b) Coefficient value of 16-N6-methyladenosine-associated lncRNA. (c) Risk score of TCGA cohort. (d) Risk score of GEO cohort. (e) Heatmap of 16 N6-methyladenosine-associated lncRNA expression in TCGA dataset. (f) Heatmap of 16 N6-methyladenosine-associated lncRNA expression in GEO dataset.

A total of 4183 lncRNAs were adopted to seek the m6A-associated factors by correlation analysis. Sixty-three lncRNAs with absolute correlation coefficients of more than 0.3 were screened out and subsequently applied to the univariate Cox hazard analysis. The univariate Cox analysis produced 19 lncRNAs related to survival, and the 19 lncRNAs were input to LASSO regression analysis for selecting the candidate variables used for model construction. Finally, 16 lncRNAs were obtained with their corresponding coefficients presented (Figures 1(a) and 1(b)). The 16 lncRNAs were used to construct an m6A score (MS) based on their expression levels and coefficients as described in Material and Method. The MS was calculated for each patient in training and validating sets, and the patients were divided into low-MS and high-MS groups according to the median MS values (Figures 1(c) and 1(d)). The differentially expressed lncRNAs ranged by the MS groups were presented in heatmaps. The high-MS and low-MS groups exhibited different expression patterns in both training and validating sets (Figures 1(e) and 1(f)).

### 3.2. Survival Analyses of the MS

**Figure 2:**
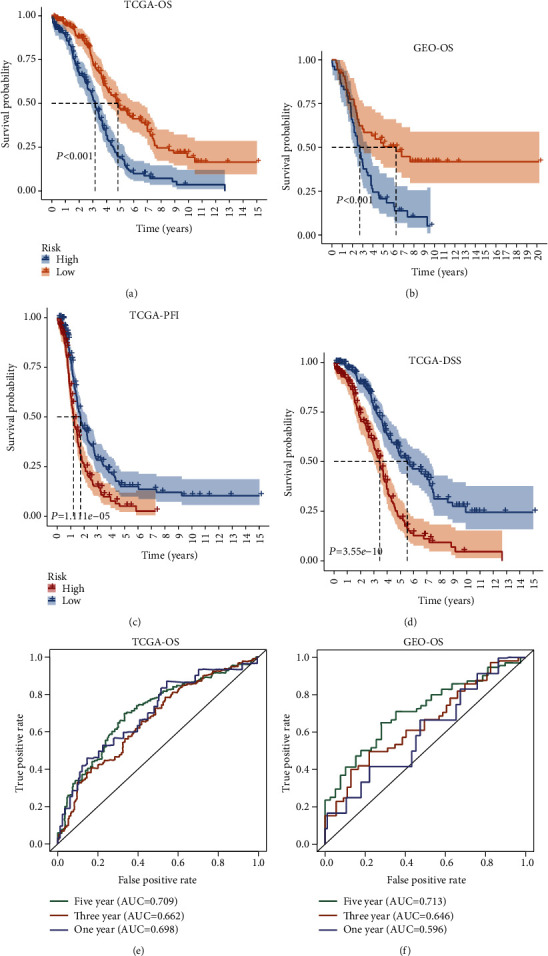
Survival analyses of the MS. (a) Kaplan-Meier survival analysis in TCGA-OS. (b) Kaplan-Meier survival analysis in GEO-OS. (c) Kaplan-Meier survival analysis in TCGA-PFI. (d) Kaplan-Meier survival analysis in TCGA-DSS. (e) ROC analysis of survival prediction at 1, 3, and 5 years in TCGA-OS. (f) ROC analysis of survival prediction at 1, 3, and 5 years in GEO-OS.

To validate the prognostic value of the MS, we conducted survival analyses by plotting Kaplan-Meier curves. As a result, the survival rate can be separated between the high-MS and low-MS groups, and the high-MS group showed low survival probabilities both in training and validating sets (Figures 2(a) and 2(b)). In addition, the MS can also predict the progress-free interval (PFI) and disease-specific survival (DSS) in TCGA dataset with the same trend as OS (Figures 2(c) and 2(d)). ROC was applied to estimate the predictive accuracy of the MS for patient survival. The area under curves (AUCs) of 5-year and 3-year overall survival (OS) prediction were high in both training (AUC = 0.709 and 0.662 for 5- and 3-year OS, respectively) and validating sets (AUC = 0.713 and 0.646 for 5- and 3-year OS, respectively). For 1-year OS, the AUC was high in the training set (0.698), while relatively lower in validating set (0.596). These results demonstrated the high prognostic value of MS in ovarian cancer patients.

### 3.3. The CNV Differences between High-MS and Low-MS Groups

**Figure 3:**
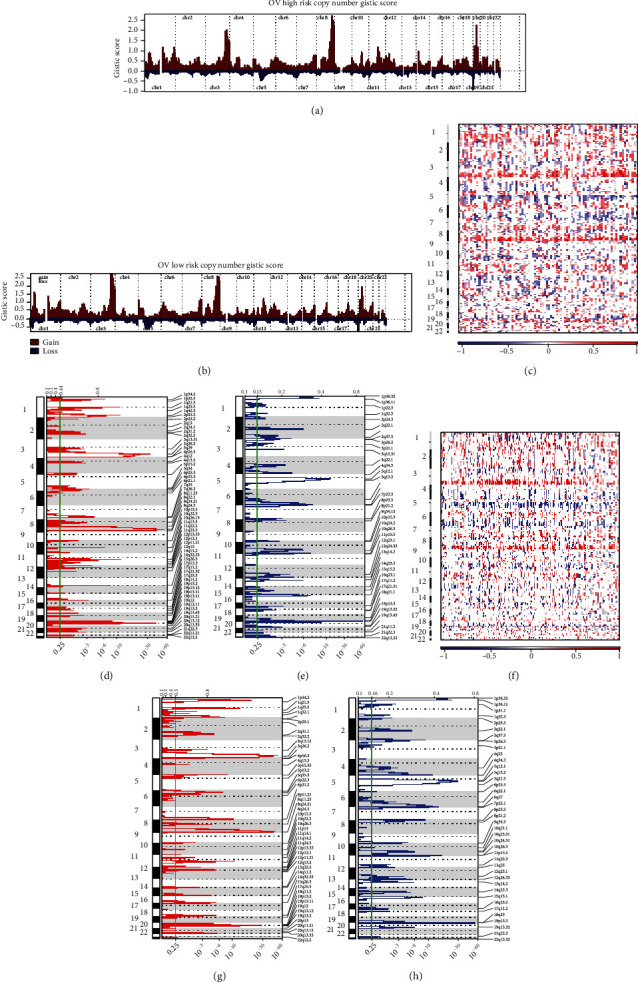
CNV differences between high-MS and low-MS groups. (a) OV high risk copy number GISTIC score. (b) OV low risk copy number GISTIC score. (c–h) The exact locations of the amplifications.

The constructed MS was capable of distinguishing the patient risks; we further explored whether patients in the two groups bore dissimilar CNV and SNP rates. The CNV consists of two forms: copy number amplifications and deletions. As exhibited in Figures 3(a) and 3(b), the high-MS patients obtained higher GISTIC scores in chromosomes 8, 10, 11, 14, 20, and 22. The exact locations of the enriched amplifications were at 8q22.1, 10q22.3, 11q22.2, 14q11.2, 20q11.21, and 22q11.21 (Figures 3(d) and 3(g)), while for copy number deletions, only 3q13.31 deletion was enriched in high-MS patients (Figures 3(e) and 3(h)). SNP rates among the high-MS and low-MS did not show dissimilarity (Supplementary Figure S1).

### 3.4. Establishment and Verification of a Clinical Nomogram

**Figure 4:**
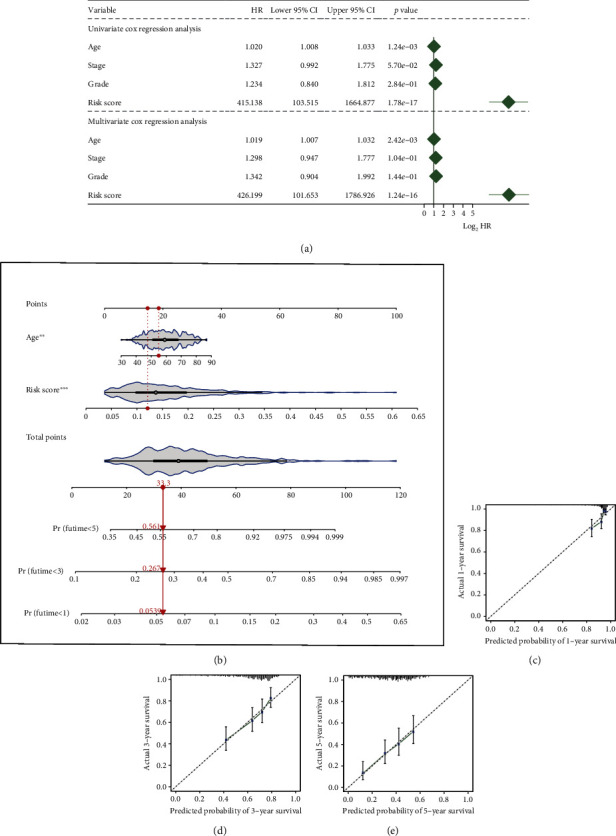
Establishment and verification of a clinical nomogram. (a) A forest plot for results of Cox regression analysis of risk score, age, stage, and grade. (b) Nomogram. (c) Calibration curves for 1 year. (d) Calibration curves for 3 years. (e) Calibration curves for 5 years.

To testify whether the MS, patient age, tumor stage, and grade can function as independent predictors for establishing a clinical nomogram, we conducted univariate and multivariate Cox regression analyses for these features. The MS and age were retained to be integrated as predictive factors (Figure 4(a)). Subsequently, the clinical nomogram was constructed based on MS and age (Figure 4(b)), and its predictive calibration for 1-, 3-, and 5-year OS was verified by calibration curves. The results showed that the nomogram predicted patient 1-, 3-, and 5-year OS with good calibration (Figures 4(c)–4(e)).

### 3.5. Functional Analyses of the m6A-Associated lncRNA Signature

**Figure 5:**
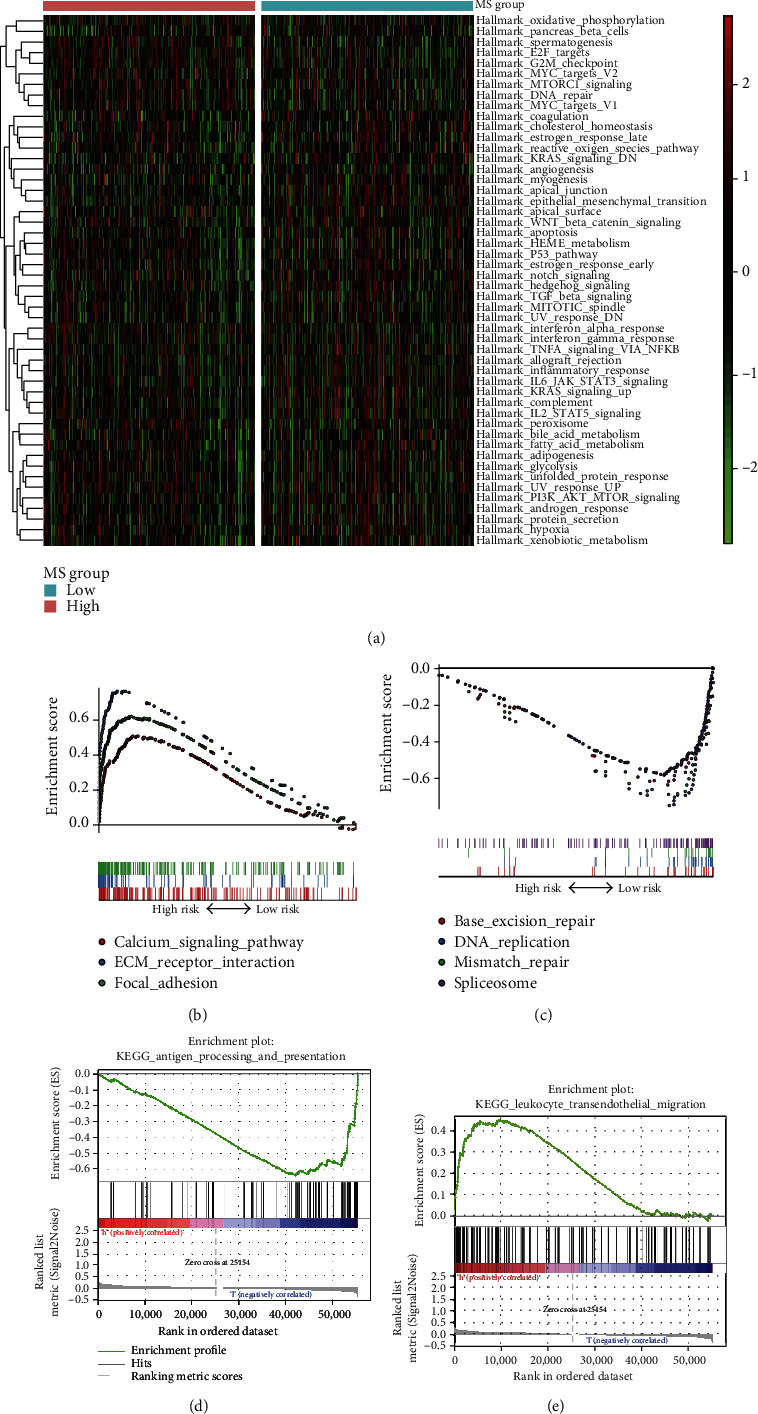
Functional analyses of the m6A-associated lncRNA signature. (a) Heatmap of pathways. (b) Negatively enriched pathways. (c) Positively enriched pathways. (d) Antigen processing and presentation pathway. (e) Leukocyte transendothelial migration.

The GSEA was performed on the 16 m6A-associated lncRNA signature, and the top 3 positively and top 4 negatively enriched KEGG pathways were presented. The positively enriched pathways include calcium, extracellular matrix (ECM) receptor, and focal adhesion signaling pathway (Figure 5(a)), and the negatively enriched pathways mainly contained the DNA repair-related pathways (Figure 5(b)), suggesting that the poor prognosis of high-MS patients was attributed to activated malignant phenotypes and genetic malfunction of cancer cells. Furthermore, we noticed that antigen processing and presentation pathway was negatively enriched (Figure 5(d)), and leukocyte transendothelial migration (Figure 5(e)) was highly enriched; this indicated the variety of immune activities in the cancer process.

### 3.6. Environment Infiltration Differences between MS Groups

**Figure 6:**
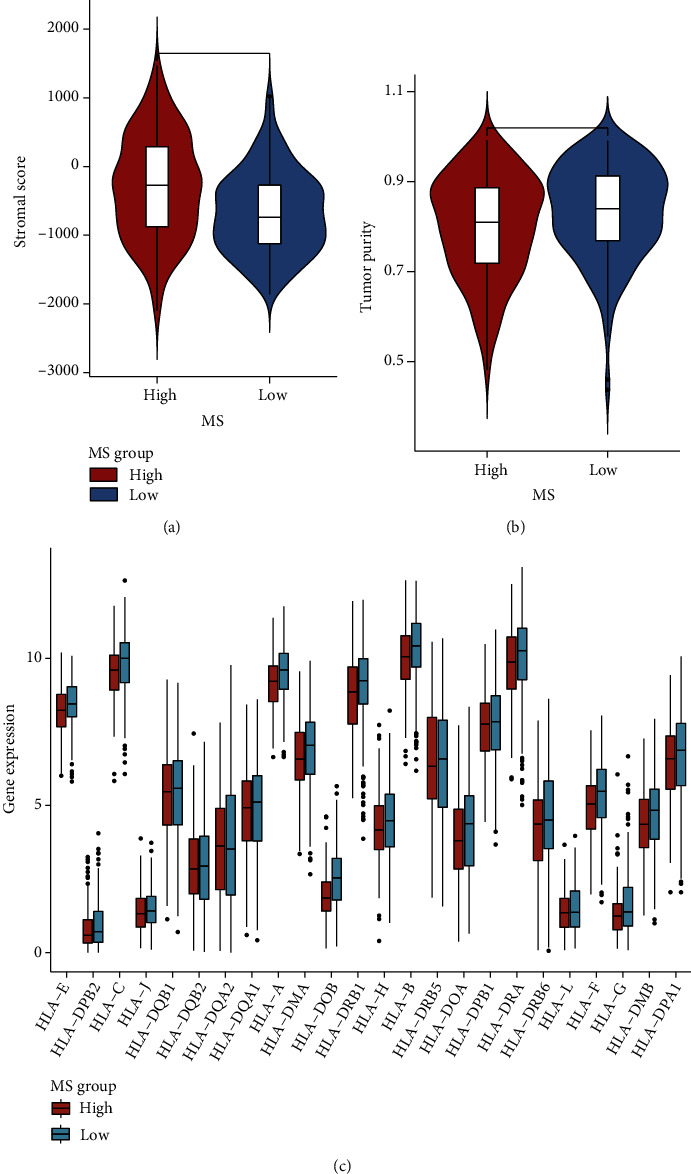
Environment infiltration differences between MS groups. (a) Differential of stromal score in the MS groups. (b) Differential of tumor purity in the MS groups. (c) Human leukocyte antigen (HLA) expressions in different MS groups.

The functional analyses have suggested the immune diversity between high-MS and low-MS groups. On top of that, we compared the tumor environment infiltrating levels between the two groups. The calculation of stromal score and tumor purity showed the high infiltration of stromal cells in the high-MS group (Figures 6(a) and 6(b)). We also estimated the immune infiltration levels of the two groups by comparing the human leukocyte antigen (HLA) expressions; the results manifested the high-MS group harbored lower HLA levels (Figure 6(c)), implying the immunocyte recruiting was suppressed in high-MS ovarian cancers.

### 3.7. MS-Related Immunocyte Infiltration

**Figure 7:**
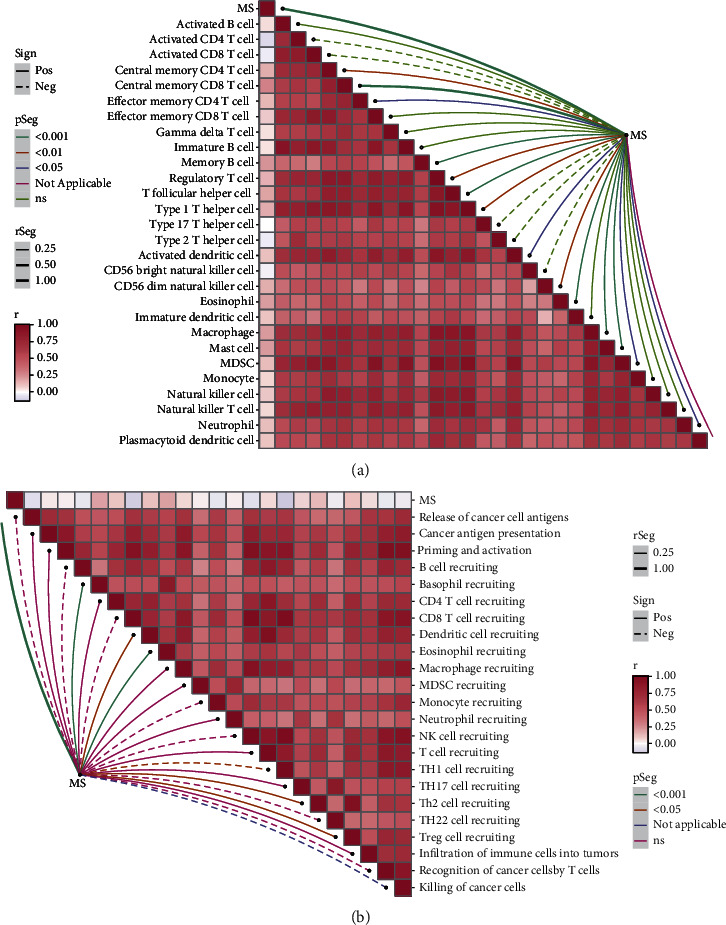
MS-related immunocyte infiltration. (a) Correlation between the immune cell content and MS. (b) Correlation between the cancer-immunity cycle and MS.

To explicit the immunocyte infiltration map, we further performed ssGSEA analyses on the MS based on 28 tumor-infiltrating lymphocytes (28 TILs) retrieved from The Cancer Immunome Atlas. As presented in Figure 7(a), the MS positively correlated to central memory CD4, CD8 T cells, effector memory CD4 cells, and memory B cells, implying the enhanced long-term adaptive antitumor immunity mediated by T cells and B cells in the high-MS group. Besides, macrophages were also observed infiltrated in the high-MS group, but their roles remain unclear. We noticed the Treg cells were also highly infiltrated in the high-MS group; this suggested an immunosuppressive role in high-MS cancers. To validate this speculation, we then conducted ssGSEA on the correlation between the cancer-immunity cycle and MS. As expected, the MS positively correlated with Th2 cell and Treg cell recruiting and negatively correlated with Th1 helper cells (Figure 7(b)). The unbalance of Th1/Th2 helper cells and recruiting of Treg cells in high-MS ovarian cancers indicated that the anticancer immunity was suppressed.

### 3.8. Immune Checkpoint and Chemotherapeutic Reaction Analyses

**Figure 8:**
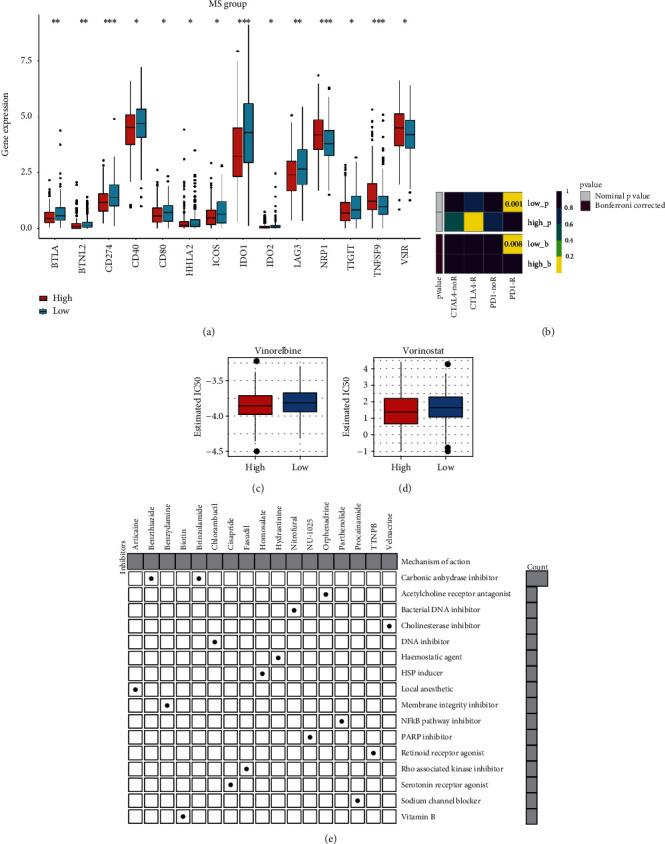
Immune checkpoint and chemotherapeutic reaction analyses. (a) Differential expression analysis of immune checkpoint in MS groups. (b) Heatmap of expression of PD1 and CTLA4. (c) IC_50_ of vinorelbine in MS groups. (d) IC_50_ of vorinostat in MS groups. (e) CMap analysis to identify the potential compounds targeting between MS groups and their corresponding mechanisms.

Since the high-MS group exhibited immunosuppressive features, we then analyzed the immune checkpoint marker levels between the two groups to seek whether immune checkpoints played roles in their immunosuppression. We noticed that the *NPR1*, *TNFSF9*, and *VSIR* genes were significantly highly expressed in the high-MS group (Figure 8(a)); the heatmap also manifested the high expression of PD1-receptor in this group (Figure 8(b)), implying the potential of immune checkpoint therapy against these markers. Besides, we compared the estimated half-maximal inhibitory concentration (IC_50_) of commonly used chemotherapy drugs, including cisplatin, vinorelbine, and vorinostat. As result, high-MS cancers presented lower estimated IC_50_ of vinorelbine and vorinostat (Figures 8(c) and 8(d)), suggesting their sensitivity to these two drugs. Finally, we applied CMap analysis to identify the potential compounds targeting the differentially expressed genes between MS groups and their corresponding mechanisms. We found that two carbonic anhydrase inhibitors were identified, including benzthiazide and brinzolamide (Figure 8(e)). These results provided promising therapeutic targets for high-MS ovarian cancers.

## 4. Discussion

In this review, we constructed a robust m6A score to predict the prognosis of ovarian cancer patients using the m6A lncRNA signature. Among various cancers, m6A lncRNA prognostic models have presented high accuracy in predicting the patients' direct prognosis or therapeutic response and in revealing the mechanism of tumor malignancy [14–16]. For robustness, our MS outperformed the current m6A risk scores for ovarian cancer survival prediction [31, 32] according to criteria for genetic model estimation [33], and our clinical nomogram also achieved high calibration for short-term or long-term survival prediction. Apart from MS, we noticed that age was also a risky factor for ovarian cancers, following the finding that the older age group has been found to harbor higher mortality [3]. This correlation demonstrated ovarian cancer an age-associated disease with the participation of m6A.

Establishment of the MS identified 12 novel m6A-related lncRNA with prognostic value, including WAC-AS1, TRAM2-AS1, SH3RF3-AS1, PCOLCE-AS1, MYCNOS, LINC01270, LINC00592, LAMTOR5-AS1, FOXN3-AS1, DLGAP1-AS2, DICER1-AS1, and ARHGAP26-AS1. WAC-AS1 was identified as a protective lncRNA in glioma [34], and LAMTOR5-AS1 was positively correlated with less aggressive prostate cancer [35]; their prognostic indications matched our discoveries, while there have been no reports concerning TRAM2-AS1, FOXN3-AS1, and ARHGAP26-AS1 in cancer studies. Hence, our study provided novel biomarkers and therapeutic targets for malignant ovarian cancer patients.

m6A has been discovered to correlate with antitumor immunity and immune evasion [36, 37] in cancers. In our study, we noticed that antigen processing and presentation pathways were blocked in the high-MS group according to the GSEA analysis, and immunocyte marker expressions were lower in the high-MS group, implying MS signature can perturb the anticancer immunity in ovarian cancer. m6A modification can control the critical pathways during differentiation of naïve T and sustained the immunosuppressive functions of Treg cells [38]. Besides, m6A “reader” IGF2BP2 switched macrophages from M1 to M2 phenotype via modulation of tuberous sclerosis 1 [39]. These discoveries were consistent with our finding that high MS was associated with high Treg cells and macrophage infiltration. Notably, the negative correlation of Th1 cells and positive correlation of Th2 cells with the MS were identified; this Th1/Th2 cell balance disruption has been previously stated to escape cancers from immune surveillance [40], and it was also distinctively detected in peripheral blood of ovarian cancer patients compared to normal patients [41], demonstrating that ovarian cancer cells harness the Th1/Th2 balance for avoiding immune attack. Comprehensive considering, the m6A signature promotes the malignancy of ovarian cancer probably by suppressing the anticancer immunity.

Since Treg cells and Th1/Th2 balance can suppress anticancer immunity via immune checkpoint-related mechanisms [42, 43], the immune checkpoint status of TCGA samples was investigated. Not surprisingly, several highly expressed checkpoint genes were observed in the high-MS group, including *NRP1*, *TNSF9*, and *VSIR*. *NRP1* targeted inhibitors were found to enhance the proliferation and antitumor effect of CD8+ T cells [44]; it was discovered as a marker for Treg cells and M2 macrophages [45, 46]. *TNSF9* was a conserved pan-cancer marker of Treg cells that affect CD8+ T cell activity [47]. *VSIR* was also found to regulate Treg cells in combination with CTLA-4 [48], as well as T2 helper cell generation and functions [49]. The discovered elevation of these checkpoint markers corresponded to our findings that high-MS has a high infiltration of Treg cells, Th2 cells, and macrophages, manifesting the m6A-associated immunosuppressive roles in ovarian cancer.

However, there are numerous limitations to our study that should be considered. Our research was only based on TCGA database, and we do not conduct vitro assays for hub lncRNAs. Beyond the effects on the immune, MS can predict the sensitivities to chemotherapeutic drugs. The low estimated IC_50_ of vinorelbine and vorinostat in the high-MS group has suggested their promising efficacy. Since no clinical trial of these two drugs' effects on ovarian cancer patients has been conducted, the future exploration of benefits from them depending on the MS status is required.

## 5. Conclusion

Comprehensively, this study established a robust tool for prognostic management of ovarian cancer patients and providing candidate adjuvant chemotherapy, as well as novel therapeutic strategies targeting the cross-talk between m6A and immunosuppression.

## Data Availability

The following information was supplied regarding data availability: data is available at TCGA (https://portal.gdc.cancer.gov/) and GEO databases (https://www.ncbi.nlm.nih.gov/geo/).

## Supplementary Materials

Supplementary Materials Figure S1: SNP rates among the high-MS and low-MS did not show dissimilarity. (A) SNP rates among the high-MS. (B) SNP rates among the low-MS.
