# Supplementary material for: m6A-Related lncRNA Signature Is Involved in Immunosuppression and Predicts the Patient Prognosis of the Age-Associated Ovarian Cancer
Source: J Immunol Res. 2022 Aug 10;2022:3258400. doi: 10.1155/2022/3258400 (PMC9385364; doi:10.1155/2022/3258400)
Supplement: Supplementary Materials — Figure S1: SNP rates among the high-MS and low-MS did not show dissimilarity. (A) SNP rates among the high-MS. (B) SNP rates among the low-MS. [file 3258400.f1.pdf]

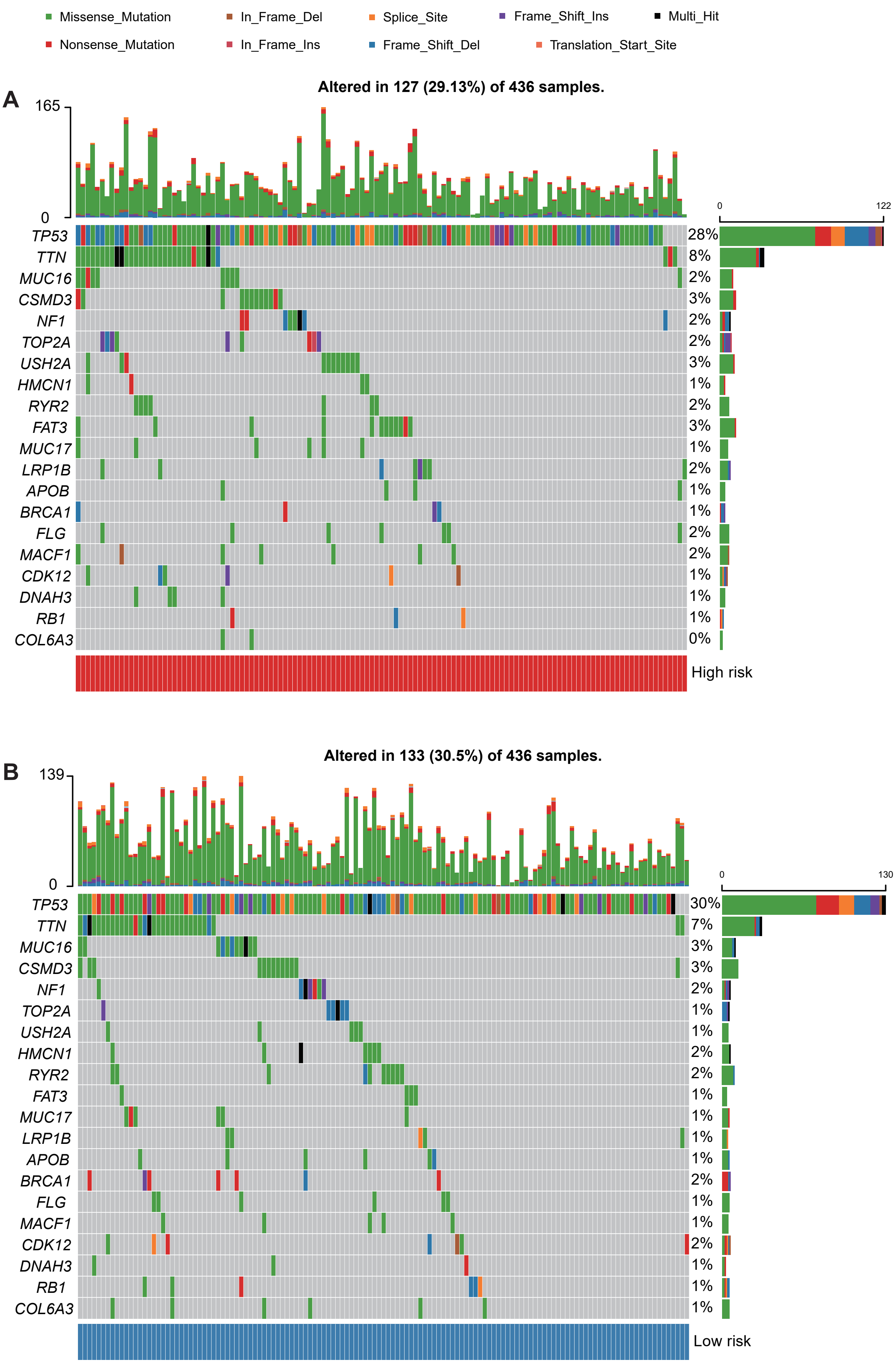

Missense_Mutation
Nonsense_Mutation
In_Frame_Del
In_Frame_Ins
Splice_Site
Frame_Shift_Del
Frame_Shift_Ins
Translation_Start_Site
Multi_Hit
A
Altered in 127 (29.13%) of 436 samples.
165
0
122
TP53 28%
TTN 8%
MUC16 2%
CSMD3 3%
NF1 2%
TOP2A 2%
USH2A 3%
HMCN1 1%
RYR2 2%
FAT3 3%
MUC17 1%
LRP1B 2%
APOB 1%
BRCA1 1%
FLG 2%
MACF1 2%
CDK12 1%
DNAH3 1%
RB1 1%
COL6A3 0%
High risk
B
Altered in 133 (30.5%) of 436 samples.
139
0
130
TP53 30%
TTN 7%
MUC16 3%
CSMD3 3%
NF1 2%
TOP2A 1%
USH2A 1%
HMCN1 2%
RYR2 2%
FAT3 1%
MUC17 1%
LRP1B 1%
APOB 1%
BRCA1 2%
FLG 1%
MACF1 1%
CDK12 2%
DNAH3 1%
RB1 1%
COL6A3 1%
Low risk
